# Supplementary material for: Chaotic Dynamics Enhance the Sensitivity of Inner Ear Hair Cells
Source: Sci Rep. 2019 Dec 5;9:18394. doi: 10.1038/s41598-019-54952-y (PMC6895040; doi:10.1038/s41598-019-54952-y)
Supplement: Supplementary file 1 — Supplemental Material [file 41598_2019_54952_MOESM1_ESM.pdf]

Supplemental Material for: Chaotic Dynamics Enhance the Sensitivity of Inner Ear Hair Cells

Justin Faber[1] and Dolores Bozovic[1,2]

[1]*Department of Physics & Astronomy and* [2]*California NanoSystems Institute, University of California, Los Angeles, California 90095, USA*

(Dated: May 8, 2019)

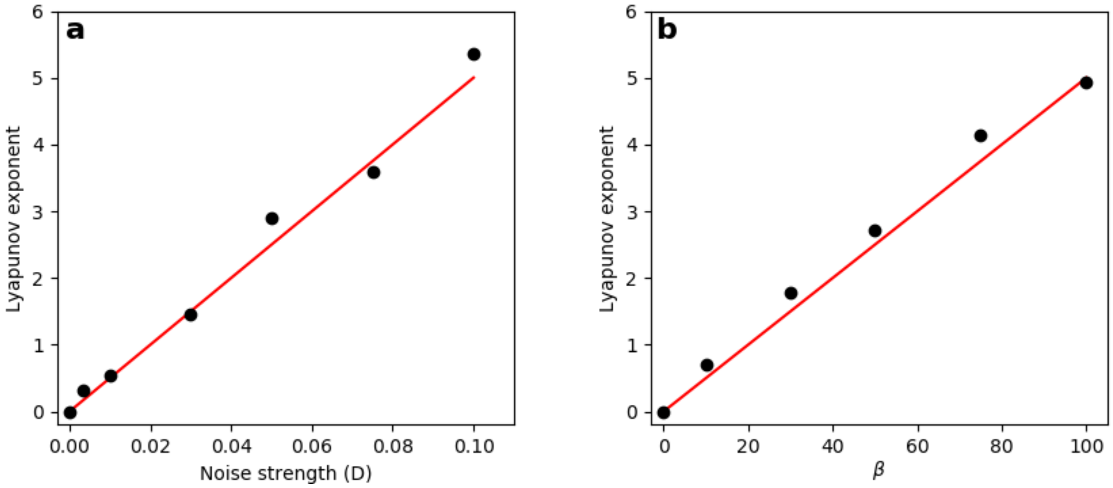

FIG. S1. Comparison of the analytic calculation of the Lyapunov exponent (red line) to the numerical calculation (black points). (**a**) The noise strength is varied, while all other parameters are fixed ($\mu = \alpha = \Omega_0 = 1$, $\beta = 50$). (**b**) β is varied, while all other parameters are fixed ($\mu = \alpha = \Omega_0 = 1$, $D = 0.05$)

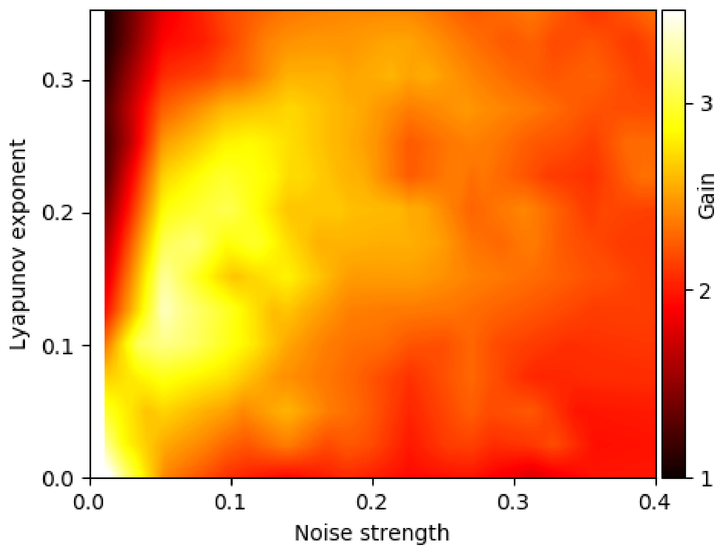

FIG. S2. Phase-locked amplitude gain for above-resonance ($\omega_{stim} = 1.05\Omega_0$), sinusoidal stimulus as the noise strength and Lyapunov exponent are varied. Color was generated by linearly interpolating a grid of 10 Lyapunov exponent values and 10 noise strengths.

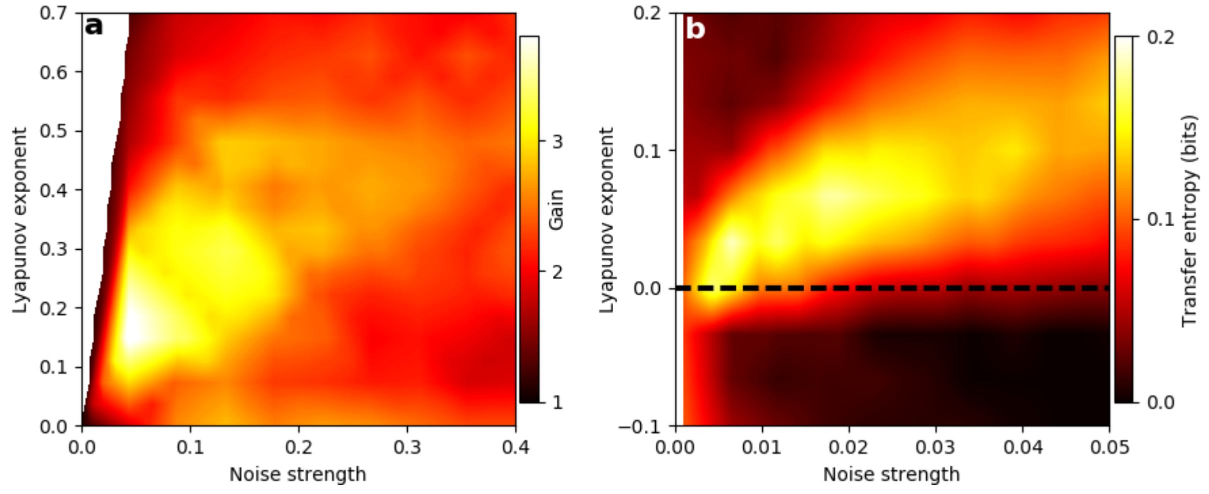

FIG. S3. (**a**) Phase-locked amplitude gain for on-resonance, sinusoidal stimulus as the noise strength and Lyapunov exponent are varied. Color was generated by linearly interpolating a grid of 10 Lyapunov exponent values and 10 noise strengths. (**b**) Transfer entropy from burst noise stimulus to response of the Hopf oscillator as noise strength and Lyapunov exponent are varied. In the $\lambda < 0$ regime, the system is quiescent, and the Lyapunov exponent characterizes the stability of this fixed point. Color was generated by linearly interpolating a grid of 10 Lyapunov exponent values and 10 noise strengths. For both panels, the Lyapunov exponent was modulated by varying μ.

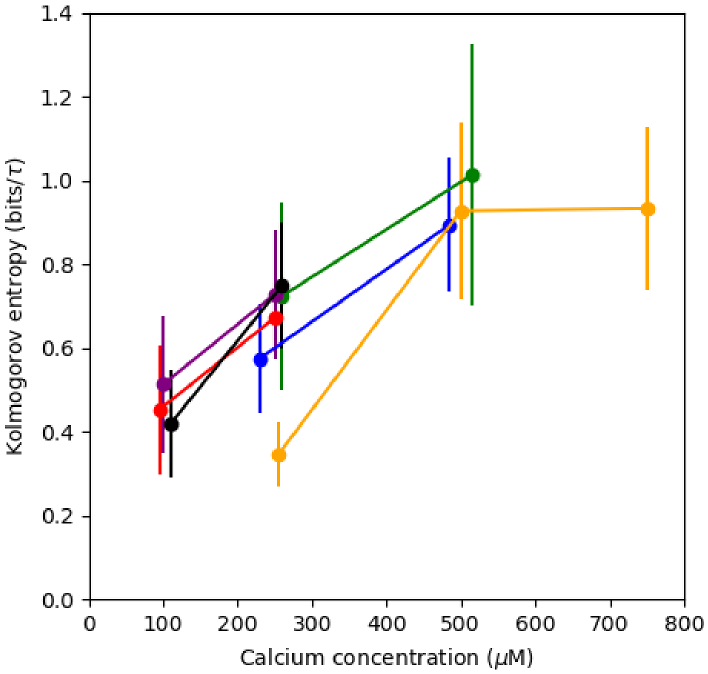

FIG. S4. The dependence of Kolmogorov entropy on the endolymph calcium concentration. Each of the six colors corresponds to a different cell.

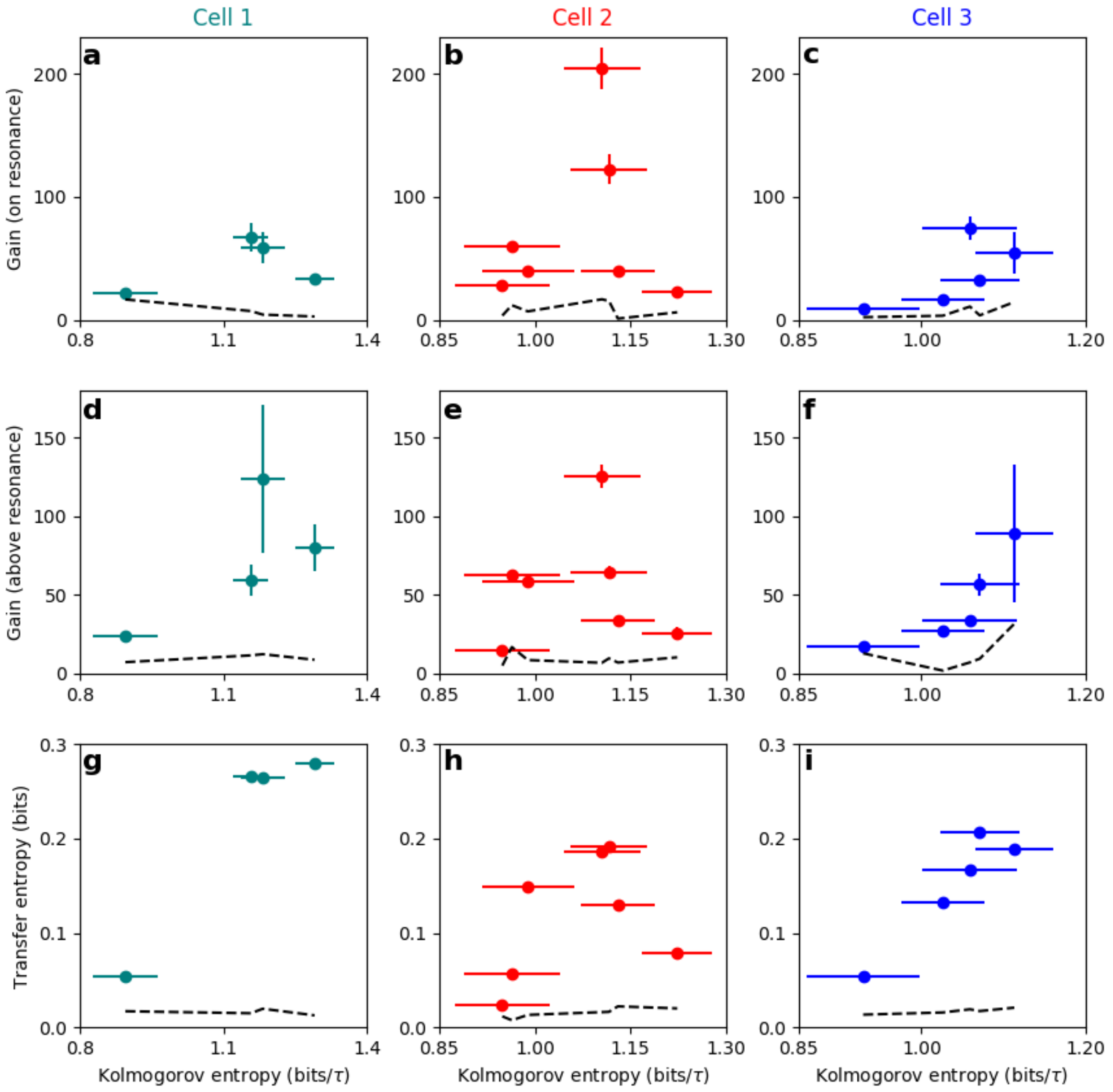

FIG. S5. Phase-locked amplitude gain for 2 pN sinusoidal stimulus at the natural frequency (**a-c**) and above the natural frequency (**d-f**) for three additional cells. Data points and error bars on the gain represent the mean and standard deviation from 100 bootstraps. The noise floor (dashed curve) was calculated by treating a segment of the spontaneous oscillation recording as if a stimulus were present and calculating the gain. This curve represents the mean plus one standard deviation from 100 bootstraps. (**g-i**) Transfer entropy from burst noise stimulus to response. Data points and error bars represent the mean and standard deviation obtained from 100 bootstraps. The noise floor (dashed curve) was determined by calculating the transfer entropy in the reverse direction (response to stimulus). This curve represents the mean plus one standard deviation from 100 bootstraps.

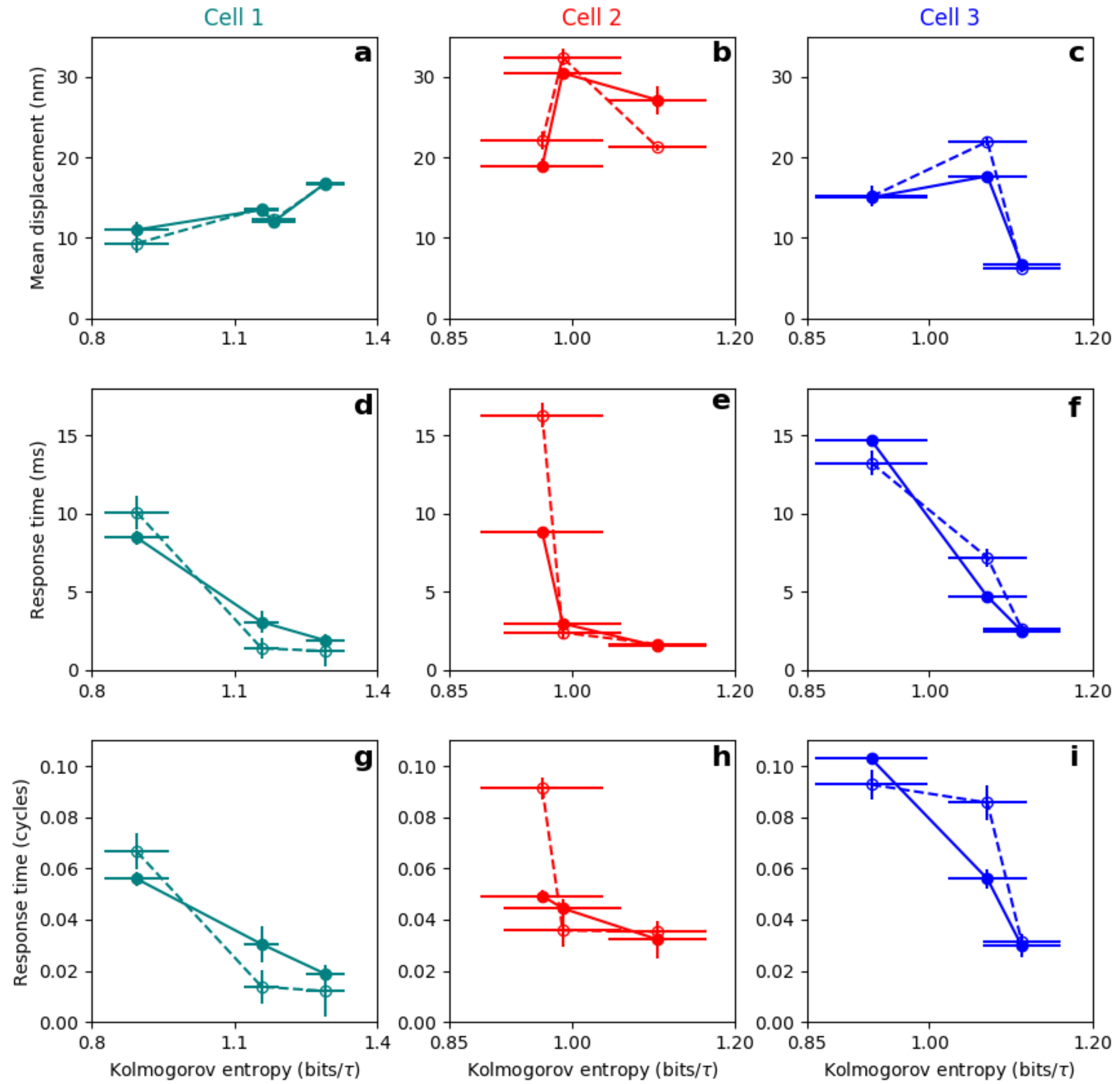

FIG. S6. (**a-c**) Average displacement induced on the hair bundle from the step stimulus, averaged over ~ 200 square waves. Data points and error bars represent the mean and standard deviation of the response plateau. (**d-f**) Response time to step stimulus, characterized by fitting the mean response to an exponential and taking the decay time. Error bars represent the standard deviation of the residual associated with the exponential fit. (**g-i**) Response times from (d-f) scaled to the natural periods of oscillation for each recording. For all panels, open-dashed and closed-solid data points represent averages over steps in the positive (channel open) and negative (channel closed) directions, respectively.
